# The Clinical Utility of D-Dimer and Prothrombin Fragment (F1+2) for Peripheral Artery Disease: A Prospective Study

**DOI:** 10.3390/biomedicines10040878

**Published:** 2022-04-11

**Authors:** Sara Arfan, Abdelrahman Zamzam, Muzammil H. Syed, Shubha Jain, Niousha Jahanpour, Rawand Abdin, Mohammad Qadura

**Affiliations:** 1 Division of Vascular Surgery, St. Michael’s Hospital, Toronto, ON M5B 1W8, Canada; saraarfan92@gmail.com (S.A.); abdelrahman.zamzam@gmail.com (A.Z.); muzammil.syed@mail.utoronto.ca (M.H.S.); jains@ucalgary.ca (S.J.); niousha.jahanpour@gmail.com (N.J.); 2 Department of Medicine, McMaster University, Hamilton, ON L8S 4K1, Canada; rawand.abdin@medportal.ca; 3 Department of Surgery, University of Toronto, Toronto, ON M5S 1A1, Canada; 4 Keenan Research Centre for Biomedical Science, Li Ka Shing Knowledge Institute, St. Michael’s Hospital, Toronto, ON M5B 1W8, Canada

**Keywords:** atherosclerosis, biomarkers, fibrinolysis, peripheral arterial disease, thrombosis

## Abstract

D-dimer and prothrombin fragment (F1+2) levels are elevated in patients with peripheral artery disease (PAD). We examined their prognostic potential in predicting decreasing ABI and major adverse limb events (MALE). A total of 206 patients were recruited from St. Michael’s Hospital and followed for two years. Baseline plasma concentrations of D-dimer and F1+2 were recorded. Pearson’s correlation was used to assess the correlation between the biomarkers and ABI at year 2. During follow-up, multivariable Cox proportional hazard analysis was performed to investigate their role in predicting decreasing ABI (defined as change in ABI > −0.15) and MALE (defined as the need for arterial intervention or major limb amputation). Cumulative survival was assessed using Kaplan–Meier analysis. Baseline D-dimer and F1+2 levels were elevated in PAD patients (median (IQR) 1.34 (0.80–2.20) for D-dimer and 3.60 (2.30–4.74) for F1+2; *p* = 0.001) compared to non-PAD controls (median (IQR) 0.69 (0.29–1.20) for D-dimer and 1.84 (1.17–3.09) for F1+2; *p* = 0.001). Both markers were negatively correlated with ABI at year 2 (r = −0.231 for D-dimer, r = −0.49 for F1+2; *p* = 0.001). Cox analysis demonstrated F1+2 and D-dimer to be independent predictors of PAD status (HR = 1.27, 95% CI = 1.15–1.54; *p* = 0.013 for D-dimer and HR = 1.28, 95% CI = 1.14–1.58; *p* = 0.019 for F1+2). Elevated baseline concentrations of D-dimer and F1+2 were associated with high incidence of decreasing ABI and 1- and 2-year event-free survival (62% and 86%, respectively). Combined analysis of D-dimer and F1+2 provides important prognostic information that facilitates risk stratification for future disease progression and MALE outcomes in patients with PAD.

## 1. Introduction

Peripheral artery disease (PAD) is a manifestation of atherosclerosis in the lower limb vasculature with a global prevalence of >200 million [1,2,3]. A substantial percentage of PAD patients remain asymptomatic and are at risk of progressing to more advanced stages presenting with intermittent claudication (IC), including chronic limb-threatening ischemia (CLTI) [4]. Ischemic symptoms are a result of vascular stenosis and thrombotic events secondary to plaque rupture [5,6]. Upon plaque rupture, the combination of hemostasis disruption and compromised endothelium leads to the release of thrombogenic proteins, which activates the coagulation cascade and results in thrombus formation [5,6]. PAD represents a hypercoagulable state, and the activation of the coagulation cascade is implicated in disease progression [7,8].

Previously, patients with CLTI were shown to be hypercoagulable and prone to thrombotic events compared to non-CLTI patients, reflecting the activation of the coagulation and fibrinolytic system [9]. Biomarkers of inflammation and endothelial injury that promote coagulation (namely, D-dimer and prothrombin fragment (F1+2)) were elevated in the CLTI cohort, indicating a directly proportional relationship between disease severity and their plasma concentrations [9], in accordance with previous studies [10].

D-dimer is a degradation product of cross-linked fibrin and reflects ongoing activation of the coagulation system. It is also a marker of fibrinolysis and is associated with thrombosis [11,12,13]. Fibrinolysis is a highly regulated process, starting with fibrin formation and the activation of the tissue plasminogen activator (t-PA) on plasminogen-binding sites. The release of tissue plasminogen activator (t-PA) from endothelial cells leads to the conversion of proenzyme plasminogen into plasmin [14]. Fibrinogen mediates the modulation of coagulation and fibrinolysis through thrombin binding, conferring antithrombin activity, and through FXIII, plasminogen, antiplasmin, and tissue-type plasminogen activator (t-PA). Fibrinogen contributes to various pathological events, including thrombosis, due to a decrease in plasma concentration of fibrinogen, its changed structural properties, or the effect of polymorphisms on clot stiffness, permeability, and resistance to lysis [15]. F1+2 is a zymogen activated by factor Xa and is a marker of thrombin activation [16]. Vidula et al. reported D-dimer levels to be independently associated with higher mortality in patients with PAD and found these levels to be highest 1–2 years before death [17]. However, the prognostic relationship between advancing PAD severity and hemostatic biomarkers has not been established to date. Herein, we investigated the predictive potential of D-dimer and F1+2 to prognosticate disease worsening and major adverse limb events (MALE) in PAD patients over a period of two years [18,19].

## 2. Materials and Methods

### 2.1. Ethics Approval

This study was approved by The Unity Health Toronto Research Ethics Board at St. Michael’s Hospital, University of Toronto, in Ontario, Canada (#16-375, 8 February 2017). Informed verbal and written consent were obtained from all patients.

### 2.2. Patient Selection

Patients presenting consecutively to ambulatory clinics at St. Michael’s Hospital between February 2018 and February 2019 were recruited for this study. As previously described, patients with PAD were defined based on an ankle-brachial index (ABI) < 0.9, as well as abnormal distal pulses with or without claudication [9,20]. A control group of patients without PAD were also recruited and defined based on an ABI ≥ 0.9, presence of palpable distal pulses, and no clinical history of claudication. In situations where the ABI could not be accurately determined due to non-compressible tibial vessels, toe-brachial index (TBI) measurements were performed. Patients with TBI < 0.7 were characterized as having PAD, whereas controls had a TBI of ≥0.7 (as per ESC and ESVM guidelines) [21,22].

Persons that met any of the following criteria were excluded from this study: (1) patients on anticoagulants, (2) patients with acute limb ischemia, (3) patients with 6-month history of acute coronary syndrome or cerebrovascular attack (CVA), (4) recent diagnosis of deep vein thrombosis, (5) patients with history/active cancer, (6) patients diagnosed with sepsis.

### 2.3. Baseline Measurements and Sample Processing

We recorded baseline demographics, history of cardiovascular diseases, cardiovascular risk factors, and smoking status as previously reported [20]. Each patient underwent lower limb arterial imaging with an ultrasound (US) and ABI/TBI as appropriate. Patients were defined as having hypercholesterolemia if they had total cholesterol > 5.2 mmol/L, triglyceride > 1.7 mmol/L, or if they were receiving lipid-lowering therapy; as having hypertension if they had a systolic blood pressure ≥ 130 mmHg, diastolic pressure ≥ 80 mm Hg, or if they were receiving blood-pressure-lowering therapy (as per AHA/ACC guidelines) [23]; and as having diabetes if they had a glycosylated hemoglobin A1c ≥ 6.5% or were taking antidiabetic medication.

Blood samples were drawn into vacutainer tubes containing sodium citrate. Plasma was prepared by immediately centrifuging each specimen at 1000× *g* for 10 min at 4 °C. Next, each sample was aliquoted and stored at −80 °C until assayed. Plasma samples that had previously been thawed were not utilized for this study.

### 2.4. Biomarkers of Thrombin Activation and ELISA

Biomarkers indicative of thrombin activation were measured in control and experimental patients as previously reported [9]. D-dimer (Thermofisher Scientific, Waltham, MA, USA) and F1+2 (MyBiosource, San Diego, CA, USA) were measured via enzyme-linked immunoassay (ELISA). Optical density was determined using a microplate reader. Duplicate assays were performed for each sample, and the optimal plasma dilution factor was identified for each tested biomarker within both assays. To minimize potential inter-assay variability, all sample analyses were carried out on the same day. Sample intra-assay coefficient of variability (CV) was <10%, while the inter-assay CV was 15%.

### 2.5. Follow-Up and Measured Outcomes

Outpatient clinic visits were scheduled at 12 and 24 months upon recruitment. During these follow-up visits, ABI, PAD symptomatic status, and PAD-related management or surgical interventions (revascularization or amputation) were assessed and recorded on a standardized data collection form. Patients undergoing a surgical procedure were seen more frequently in the clinic as per the primary surgeon. The primary outcome of this study was incidence of decrease in ABI in patients during the follow-up period, defined as change in ABI ≥ −0.15 from baseline [3,24]. Secondary outcome included the incidence of major adverse limb events (MALE), defined as composite of PAD-specific outcomes such as severe limb ischemia leading to an arterial intervention (open or endovascular revascularization) or major vascular amputation (above the level of the ankle) [25]. Outcomes are reported as cumulative incidences (proportion of patients experiencing an event) and incidence rates (events/100 person-years (PYs)).

### 2.6. Statistical Methods

Demographics and clinical characteristics are expressed as mean and standard deviations (SDs) or percentages (%). Continuous data were compared using Student’s *t*-test or ANOVA if conforming to normal distribution; otherwise, Mann–Whitney U test was used. Chi-square test was used to compare categorical variables. F1+2 (nmol/mL) and D-dimer (μg/mL) levels are expressed as medians with interquartile ranges (IQRs).

Event rates at 24 months were calculated for decreasing ABI (change in ABI ≥ −0.15), need for arterial intervention, major limb amputation, and MALE. Cox proportional hazard analysis was performed to determine the ability of F1+2 and D-dimer to predict changes in ABI (≥−0.15) and MALE events for the entire study cohort. Variables that were deemed confounders (age (in years)), sex (male vs. female), hypertension (yes vs. no), hyperlipidemia (yes vs. no), smoking (yes vs. no), diabetes (yes vs. no), and history of coronary artery disease (CAD) (yes vs. no)) were included in the multivariate analysis. For the Kaplan–Meier curves, medians of D-dimer and F1+2 levels were used as cut-off points. The event-free curves were computed, and comparison of event-free survival between groups was performed using the log-rank test. Lastly, subgroup analysis was conducted to clinically risk-stratify patients based on their median F1+2 and D-dimer levels. Thus, the median level served as the reference point; patients with levels above the median were categorized as having high F1+2 and/or D-dimer levels, whereas patients with levels below the median were categorized as having low F1+2 and/or D-dimer levels. To that end, patients were divided into four groups: (1) normal group; (2) high F1+2 group; (3) high D-dimer group; and (4) high F1+2 + high D-dimer group. Statistical significance was established at *p* < 0.05 (two-sided). SPSS software, version 23 (SPSS Inc., Chicago, IL, USA), was used for data entry and statistical analysis. Microsoft Excel was used for graphical illustrations.

## 3. Results

### 3.1. Overall Characteristics and Study Cohorts

A total of 206 patients overall were enrolled in this study. Patients had a mean (SD) age of 69 years (10) and were mostly males (68%). Prevalent comorbidities included hypertension (72%), hyperlipidemia (75%), and diabetes (35%), among others (Table 1). Median (IQR) plasma levels of D-dimer and F1+2 were 1.20 μg/mL (0.67–2.01) and 3.25 nmol/mL (1.85–4.37), respectively.

Patients were stratified based on their disease status into PAD (*n*, % = 163, 79%) and non-PAD groups (*n*, % = 43, 21%). The baseline characteristics and clinical characteristics of these groups are compared in Table 1. Hyperlipidemia and diabetes were significantly higher in the PAD group as compared to the non-PAD group (*p* = 0.048 and 0.001, respectively). Baseline median (IQR) levels of F1+2 were significantly higher in the PAD group relative to the non-PAD group (3.60 [2.30–4.74] vs. 1.84 [1.17–3.09], *p* = 0.001) and can be found in Table S1. Similarly, median (IQR) D-dimer levels were significantly higher for the PAD group as compared to the non-PAD group (1.34 [0.82–2.27] vs. 0.70 [0.30–0.925], *p* = 0.001) (see Table S1).

### 3.2. Clinical Outcomes

Follow-up was available for 90.8% of patients, with a mean (SD) duration of 21.6 (3.9) months. Over the two-year follow-up period, 53 patients (26%) had an ABI drop of ≥0.15, 25 patients (12%) required an arterial intervention, 5 (2%) underwent limb loss, and 29 patients (14%) had MALE outcomes. Event rates of change in ABI, the need for arterial intervention, limb loss events, and MALE were significantly higher in the PAD group as compared to the non-PAD group (Table 1). A decrease in ABI occurred in 26% (12.86/100 PYs) of patients, 29% (14.72/100 PYs) of patients with PAD and 12% (5.81/100 PYs) in patients without a prior history of PAD (see Table S2). Regarding patients without PAD, 5 events of decreasing ABI were recorded. All of these patients had an initial baseline ABI of 1.2–1.1, but when measured again at the end of the study, their ABI was found to be in the normal range (above 0.9).

Cox analysis was only conducted for one outcome (decrease in ABI) and not the other outcomes (need for arterial intervention, amputation, and MALE), as they had low event rates (due to our findings, reports that ABI change ≥−0.15 had a significant hazard ratio (HR) for both F1+2 and D-dimer (HR 1.33 [95% CI: 1.19–1.61]; *p* = 0.004, HR 1.28 [95% CI: 1.16–1.53]; *p* = 0.008, respectively). To account for several potential confounders, we then performed multivariable Cox proportional hazard modeling, adjusting for age, sex, hypertension, hyperlipidemia, smoking, diabetes, and history of CAD. Our results indicated that ABI change ≥−0.15 remained statistically significant for F1+2 and D-dimer (HR 1.28 [95% CI: 1.14–1.58]; *p* = 0.019, HR 1.27 [95% CI: 1.15–1.54]; *p* = 0.013, respectively) (Table 2).

### 3.3. Survival Analysis and Risk Stratification Using a Combination of F1+2 and D-Dimer

**Figure 1:**
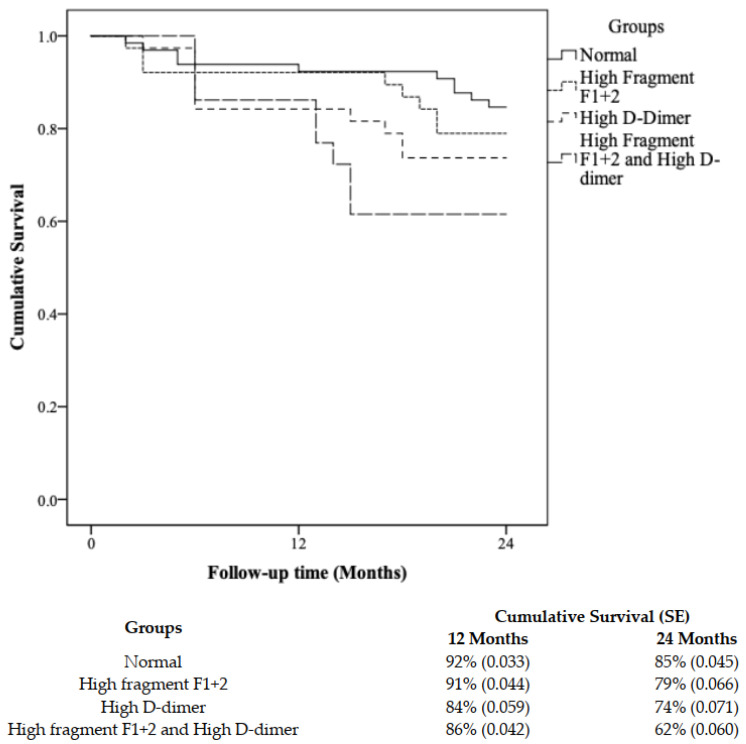
Kaplan–Meier analysis for ABI change ≥ −0.15 in patients with peripheral artery disease (PAD) stratified into four subgroups based on the F1+2 and D-dimer median values. Subgroup classification: normal (*n* = 65, F1+2 ≤ 3.25 nmol/mL and D-Dimer ≤ 1.20 μg/mL); high fragment F1+2 (*n* = 38, F1+2 > 3.25 nmol/mL and D-dimer ≤ 1.20 μg/mL); high D-dimer (*n* = 38, F1+2 ≤ 3.25 nmol/mL and D-dimer > 1.20 μg/mL); high fragment F1+2 and D-dimer (*n* = 65, F1+2 > 3.25 nmol/mL and D-dimer > 1.20 μg/mL).

To aid with clinical risk stratification of PAD patients, all patients were stratified into four groups based on median levels of F1+2 and D-dimer: (1) normal group (*n* = 65): F1+2 ≤ 3.25 nmol/mL and D-dimer ≤ 1.20 μg/mL; (2) high F1+2 group (*n* = 38): F1+2 > 3.25 nmol/mL and D-dimer ≤ 1.20 μg/mL; (3) high D-dimer group (*n* = 38): F1+2 ≤ 3.25 nmol/mL and D-dimer > 1.20 μg/mL; and (4) high F1+2 + high D-dimer group (*n* = 65): F1+2 > 3.25 nmol/mL and D-dimer > 1.20 μg/mL. At two years, we found the clinical characteristics and cardiovascular risk factors to be the same across all four subgroups except for PAD and history of CAD (*p* [trend] = 0.001 and 0.042, respectively) (Table 3). The event rates for ABI change and MALE were highest in group 4 (high F1+2 + high D-dimer), with ABI change being significant (*p* < 0.05). Over the two-year study period, our data show that high levels of F1+2 and D-dimer can significantly stratify patients with the risk of change in ABI (*p* = 0.019). The 1- and 2-year event-free survival (change ≥−0.15) for the high F1+2 and D-dimer group was 86% and 62%, respectively, as compared to the high F1+2 group (91% and 79%), high D-dimer group (84% and 74%), and normal group (92% and 85%) (Figure 1).

## 4. Discussion

In the present study, we found F1+2 and D-dimer concentrations at baseline to be elevated in patients with PAD in comparison to patients without PAD. Relative to patients without PAD, as anticipated, patients with PAD suffered more from PAD disease progression as well as MALE. Lastly, the combined measurement of F1+2 and D-dimer at baseline may be utilized for the risk stratification of patients for decreasing ABI.

The combination of F1+2 and D-dimer at baseline successfully predicted worsening PAD status at 24 months. Patients with high levels of F1+2 and D-dimer had decreased survival rates. Based on our data, the combined measurement of F1+2 and D-dimer at admission may be a novel risk stratification method for predicting future ABI (change ≥−0.15) events. As far as the authors know, the present study is the first to utilize a combination of hemostatic biomarkers to accurately diagnose and risk-stratify PAD patients for both worsening disease status and the onset of future MALE outcomes over a two-year period. Notably, subgroup analysis revealed the presence of PAD and a history of CAD to be independent risk factors for both ABI events and MALE. As such, concomitant vascular disease (PAD and CAD) directly reflects the extent and the severity of the PAD process. This finding has clinical implications for PAD patients who are at high risk for future vascular events.

To date, no single or combination of biomarkers has been used for clinical risk stratification in patients with PAD. The present study highlights the potential clinical utility of F1+2 and D-dimer for managing PAD patients and offers clinicians a biomarker-supported strategy to implement a personalized targeted regimen to prevent future worsening of disease status, morbidity, and overall mortality in PAD patients. The COMPASS trial (Cardiovascular Outcomes in People using Anticoagulation Strategies) highlighted hypercoagulable states such as PAD to be associated with a significant cardiovascular risk and indicated that a combination of low-dose anticoagulant and anti-platelet can improve cardiovascular outcomes and reduce mortality in PAD patients [25]. Our study did not investigate whether the investigated biomarkers are involved in the initial disease pathogenesis; however, we monitored the development of vascular events (ABI events and MALE) over time, as these biomarkers are related to thrombotic events that result in clinically evident PAD progression [10]. Management studies are required to test the utility of this combination for individual risk stratification.

Our data demonstrated higher levels of hemostatic biomarkers in patients with PAD, in accordance with other previous studies [26]. Furthermore, our findings demonstrate that these biomarkers can reliably predict the onset of future arterial events over a two-year period, in agreement with previous studies that demonstrated D-dimer to be a predictor of PAD deterioration and subsequent thrombotic events [27,28]. In stark contrast, however, Boneu et al. suggested that D-dimer cannot significantly predict arterial events over a two-year follow-up period [29]. The disparity behind these findings could be attributed to the multifactorial mechanisms responsible for thrombin generation and its manifold triggers, some of which are present briefly, whereas others are present over a prolonged period. Moreover, PAD patients within 2 months of an ischemic heart disease event were also reported to have increased D-dimer levels [12]. This may be because transiently elevated D-dimer levels potentially indicate the remodeling of atherosclerotic plaque [8]. However, the predictive potential of D-dimer in PAD patients for long-term follow-up needs to be ascertained, as D-dimer appears to lose its independent association after adjusting for confounders or after increasing follow-up [30,31]. This effect has been described by many others who classified D-dimer as a better predictor for short-term (1–3 years) thrombotic events and cardiovascular mortality as opposed to long-term [8,12].

Our study has certain limitations that warrant mentioning. First, a small proportion of patients (*n* = 31) were lost to follow-up due to COVID-related restrictions that prevented in-person ABI measurements. Second, follow-up was limited to two years. The relatively small sample size and short follow-up period may result in potential bias. Thus, future studies should aim for a longer follow-up period with a larger sample size to demonstrate associations that are more precise. Lastly, to closely monitor whether levels of D-dimer show a steady increase leading up to an ischemic (CTLI) or ABI event, more frequent sampling is required.

## 5. Conclusions

In summary, our study revealed for the first time that the combined measurement of F1+2 and D-dimer at admission provides reliable risk stratification for future disease worsening and adverse vascular outcomes in patients with PAD. However, it remains unclear whether F1+2 and D-dimer play a causal role in the pathophysiology of adverse events (ABI events and MALE) or whether they simply mark the extent of the disease and are therefore related to the event.

## Figures and Tables

**Table 1:** Patient demographics and clinical characteristics.

Variable	Overall *n* = 206 (%)	Non-PAD *n* = 43 (%)	PAD *n* = 163 (%)	*p*-Value ^α^
***Demographics and Clinical Characteristics at Baseline***				
**Mean (SD) ^‡^**				
ABI	0.71 (0.23)	1.04 (0.07)	0.62 (0.16)	0.001 *
Age, years	69 (10)	67 (12)	69 (10)	0.249
**N (%) ^¶^**				
Sex, male	141 (68)	32 (74)	109 (67)	0.365
Hypertension	148 (72)	29 (67)	119 (74)	0.448
Hyperlipidemia	152 (75)	27 (63)	125 (78)	0.048 *
Diabetes	72 (35)	4 (9)	68 (42)	0.001 *
Smoking, current	63 (31)	10 (23)	53 (33)	0.213
Smoking, past	113 (55)	24 (56)	89 (55)	0.213
History of congestive heart failure	7 (3)	1 (2)	6 (4)	0.670
History of coronary artery disease	71 (35)	11 (26)	60 (38)	0.205
History of stroke	28 (14)	6 (14)	22 (14)	0.938
***Vascular events at 2-year follow-up***				
**N (%) ^¶^**				
Decrease in ABI (≥−0.15)	53 (26)	5 (12)	48 (29)	0.017 *
Arterial intervention	25 (12)	0 (0)	25 (15)	0.006
Major limb amputation	5 (2)	0 (0)	5 (3)	NA
MALE	29 (14)	0 (0)	29 (18)	0.003 *

Abbreviation: *ABI*, ankle-brachial index; *MALE*, major adverse limb event. ^α^ The significance of the difference between PAD and non-PAD groups. * Significant differences are not with an asterisk. ^‡^ Differences between groups were compared using Student’s *t*-test. ^¶^ Differences between groups were compared using chi-square test.

**Table 2:** Univariate and multivariate Cox proportional hazard analysis of clinical events for F1+2 (nmol/mL) and D-dimer (μg/mL) at two-year follow-up.

	F1+2 nmol/mL				D-Dimer μg/mL			
Clinical Event	Unadjusted HR (95% CI)	*p*-Value	Adjusted HR ^‡^ (95% CI)	*p*-Value	Unadjusted HR (95% CI)	*p*-Value	Adjusted HR ^‡^ (95% CI) *	*p*-Value
Change in ABI (≥−0.15)	1.33 (1.19–1.61)	0.004	1.28 (1.14–1.58)	0.019	1.28 (1.16–1.53)	0.008	1.27 (1.15–1.54)	0.013

Abbreviations: *ABI*, ankle-brachial index; *MALE*, major adverse limb event. * Significant differences are not with an asterisk. ^‡^ Adjusted hazard ratios after adjusting for age, sex, hypertension, hyperlipidemia, smoking, diabetes, and history of CAD.

**Table 3:** Clinical characteristics of subgroups based on the median values of F1+2 and D-dimer.

Clinical Characteristics	Group 1: Normal (*n* = 65)	Group 2: High F1+2 (*n* = 38)	Group 3: High D-Dimer (*n* = 38)	Group 4: High and D-Dimer (*n* = 65)	*p* (Trend)
**Mean (SD) ^‡^**					
Age, years	69 (11)	68 (10)	67 (10)	68 (10)	0.856
**N (%) ^¶^**					
Peripheral artery disease	39 (60)	31 (82)	29 (76)	64 (99)	0.001
Sex, male	44 (68)	25 (66)	30 (79)	42 (65)	0.092
Hypertension	43 (66)	30 (79)	28 (74)	47 (72)	0.562
Hyperlipidemia	43 (66)	31 (82)	30 (79)	48 (74)	0.299
Diabetes	22 (34)	12 (32)	15 (40)	23 (35)	0.903
Smoking, current	18 (28)	14 (37)	12 (32)	19 (29)	0.888
History of congestive heart failure	1 (2)	1 (3)	2 (5)	3 (5)	0.694
History of coronary artery disease	16 (25)	16 (42)	19 (50)	20 (31)	0·042
History of stroke	10 (15)	6 (16)	2 (5)	10 (15)	0.0431
**Event Rates**					
ABI change (≥−0.15) event	10 (15)	8 (21)	10 (26)	25 (26)	0.022
Arterial intervention	5 (8)	4 (11)	7 (18)	9 (14)	0.412
Major limb amputation	1 (2)	1 (3)	1 (3)	2 (3)	0.951
MALE	6 (9)	5 (13)	8 (21)	10 (15)	0.406

Abbreviations: *ABI*, ankle-brachial index; *MALE*, major adverse limb event. ^‡^ Differences between the subgroups were compared using ANOVA. ^¶^ Differences between the subgroups were compared using the chi-square test.

## Data Availability

All data generated or analyzed during this study are included in this published article (and its Supplementary Information files).

## Supplementary Materials

The following supporting information can be downloaded at: https://www.mdpi.com/article/10.3390/biomedicines10040878/s1, Table S1. Baseline protein levels between the PAD and non-PAD control group; Table S2. Vascular outcomes as cumulative incidences (proportion of patients experiencing an event) and incidence rates (events/100 person-years [PYs]). The supporting information is included in the manuscript. Any supporting data are available upon request.
