# Supplementary material for: The Clinical Utility of D-Dimer and Prothrombin Fragment (F1+2) for Peripheral Artery Disease: A Prospective Study
Source: Biomedicines. 2022 Apr 11;10(4):878. doi: 10.3390/biomedicines10040878 (PMC9028031; doi:10.3390/biomedicines10040878)
Supplement: Supplementary file 1 [file biomedicines-10-00878-s001.zip › biomedicines-1626760-supplementary.pdf]

## Supplementary Materials

**Table S1.** Baseline protein levels between the PAD and non-PAD control group.

| Proteins (at Baseline) | Non-PAD ($n$ = 43) | PAD ($n$ = 163) | $p$ |
|---|---|---|---|
| F1+2 (nmol/mL) | 1.84 (1.17–3.09) | 3.60 (2.30–4.74) | 0.001 |
| D-dimer (μg/mL) | 0.696 (0.298–1.20) | 1.34 (0.817–2.27) | 0.001 |

**Table S2.** Vascular outcomes as cumulative incidences (proportion of patients experiencing an event) and incidence rates (events/100 person-years [PYs]).

| Vascular Events | OVERALL<br>Events/100PYs | NON-PAD<br>Events/100PYs | PAD<br>Events/100PYs |
|---|---|---|---|
| Change in ABI (≥−0.15) | 12.86 | 5.81 | 14.72 |
| Arterial Intervention | 6.07 | 0.00 | 7.67 |
| Major Limb Amputation | 1.21 | 0.00 | 1.53 |
| MALE | 7.04 | 0.00 | 8.90 |
